# Lipids: Emerging Players of Microglial Biology

**DOI:** 10.1002/glia.24654

**Published:** 2024-12-17

**Authors:** Priya Prakash, Caitlin E. Randolph, Katherine A. Walker, Gaurav Chopra

**Affiliations:** ^1^ Department of Chemistry Purdue University West Lafayette Indiana USA; ^2^ Neuroscience Institute, NYU Grossman School of Medicine New York New York USA; ^3^ Purdue Institute for Integrative Neuroscience, Purdue University West Lafayette Indiana USA; ^4^ Purdue Institute for Drug Discovery, Purdue University West Lafayette Indiana USA; ^5^ Purdue Institute of Inflammation, Immunology and Infectious Disease, Purdue University West Lafayette Indiana USA; ^6^ Regenstrief Center for Healthcare Engineering, Purdue University West Lafayette Indiana USA

**Keywords:** fatty acids, inflammation, lipid droplets, lipidomics, lipids, microglia, phospholipids

## Abstract

Lipids are small molecule immunomodulators that play critical roles in maintaining cellular health and function. Microglia, the resident immune cells of the central nervous system, regulate lipid metabolism both in the extracellular environment and within intracellular compartments through various mechanisms. For instance, glycerophospholipids and fatty acids interact with protein receptors on the microglial surface, such as the Triggering Receptor Expressed on Myeloid Cells 2, influencing cellular functions like phagocytosis and migration. Moreover, cholesterol is essential not only for microglial survival but, along with other lipids such as fatty acids, is crucial for the formation, function, and accumulation of lipid droplets, which modulate microglial activity in inflammatory diseases. Other lipids, including acylcarnitines and ceramides, participate in various signaling pathways within microglia. Despite the complexity of the microglial lipidome, only a few studies have investigated the effects of specific lipid classes on microglial biology. In this review, we focus on major lipid classes and their roles in modulating microglial function. We also discuss novel analytical techniques for characterizing the microglial lipidome and highlight gaps in current knowledge, suggesting new directions for future research on microglial lipid biology.

## Main Points

- Lipids are crucial in mediating microglial metabolism and function.
- The microglial lipidome is vast and complex, with over ten thousand lipid species.
- We discuss key lipid classes and their roles in microglial biology with insights into future work.

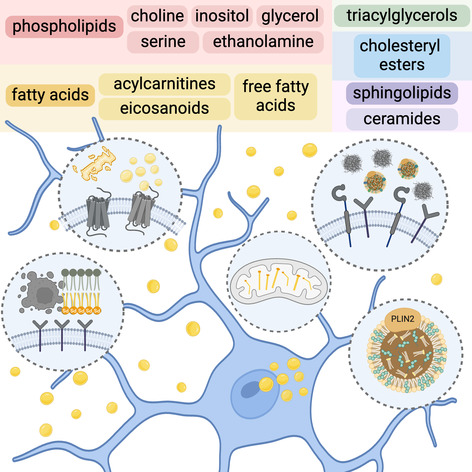

## 1 Introduction

Microglia are the resident immune cells of the central nervous system (CNS) that play critical roles in development and disease (Li and Barres 2018; Aguzzi, Barres, and Bennett 2013). They originate from the yolk sac during early embryonic development and migrate to the brain, where they permanently reside, making up 10%–15% of the total brain cell population (Alliot, Godin, and Pessac 1999). In a healthy brain, homeostatic microglia exhibit a ramified morphology with elongated processes that constantly survey their environment for external cues, such as pathogens, cellular debris, and misfolded protein aggregates, for phagocytic uptake (Nimmerjahn, Kirchhoff, and Helmchen 2005; Prakash et al. 2021). However, when triggered by an inflammatory insult, microglia transform into a reactive state, characterized by an amoeboid or dystrophic morphology, undergoing dramatic phenotypic, molecular, and functional changes (Bachstetter et al. 2015). In addition to phagocytosis, microglia perform a variety of functions, including the secretion of cytokines (Smith et al. 2012) and other immunomodulatory molecules, such as lipids and metabolites (Folick, Koliwad, and Valdearcos 2021). These functions facilitate the pruning of neuronal synapses and modulation of neuronal connectivity (Hong, Dissing‐Olesen, and Stevens 2016), which are essential for maintaining tissue homeostasis.

**FIGURE 1:**
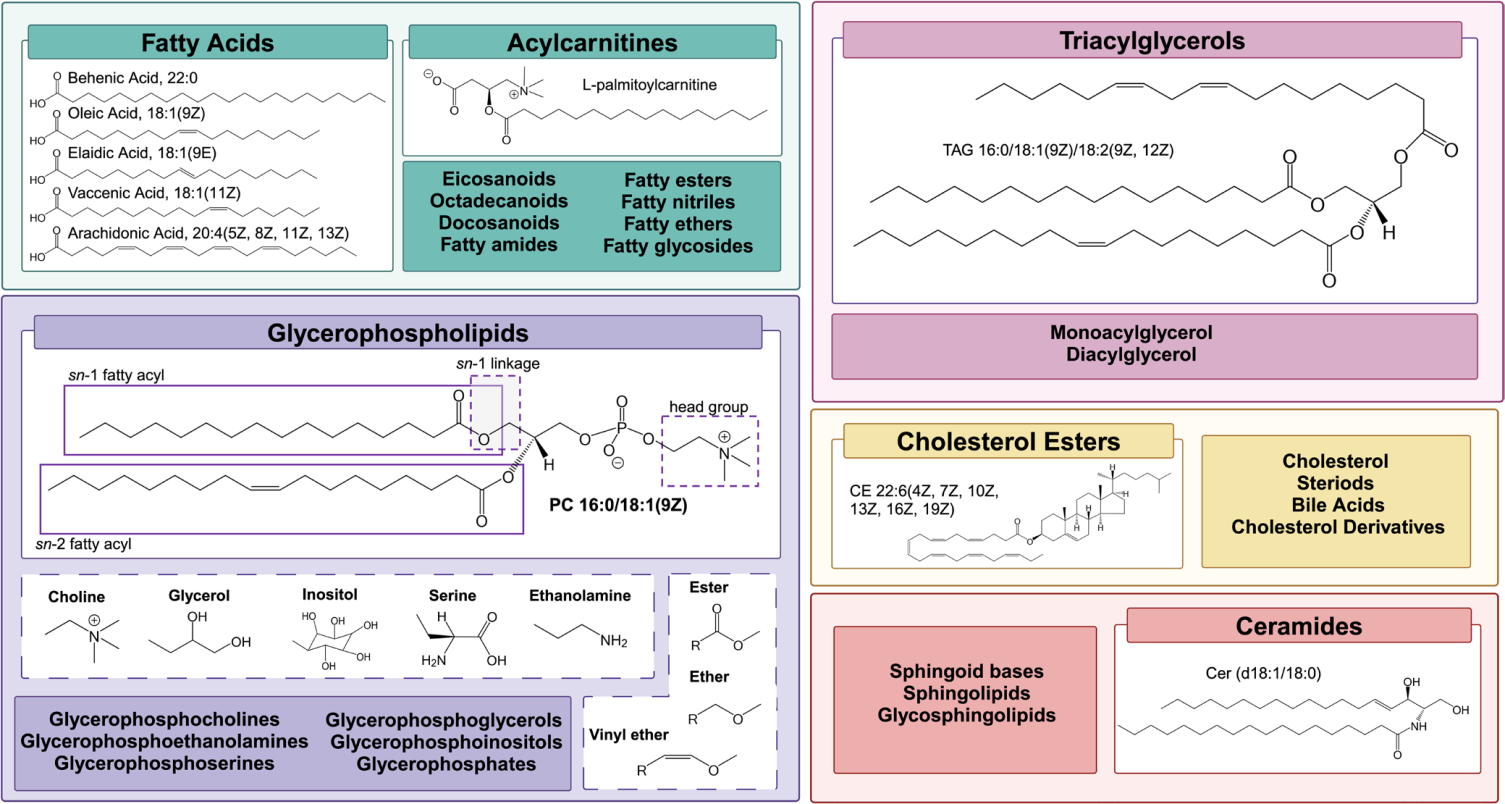
Overview of lipid classification and structural diversity. Lipids are broadly categorized into eight categories per the International Lipid Classification and Nomenclature Committee: Fatty acyls (FA—shown in turquoise), glycerolipids (GL—shown in pink), glycerophospholipids (GP—shown in purple), sphingolipids (SP—shown in red), sterols (ST—shown in yellow), prenol lipids (PR), saccharolipids (SL), and polyketides (PK). Within each main lipid category exist additional lipid classes. For example, the glycerolipid class (highlighted in pink) contains the triacylglycerol, diacylglycerol, and monoacylglycerol lipid subclasses. Similarly, glycerophospholipids (GPs) contain a number of classes characterized by polar head group composition. In the purple panel (bottom left), example *sn*‐1 radyl chains are portrayed, noting that the nature of the *sn*‐1 linkage dictates GP subclass. Explicitly, GP contain either an acyl, 1‐*O*‐alkyl, or a 1‐*O*‐alk‐1′‐enyl group at the *sn*‐1 position, indicating the diacyl, plasmanyl, or plasmenyl subclasses, respectively. Noting that lipid species within each major lipid class and subclass can exhibit hundreds, if not thousands, of structural features dictated by the presence and position of double bonds, functional groups, length of acyl chains, and so on, the lipidome is highly complex and diverse.

**FIGURE 2:**
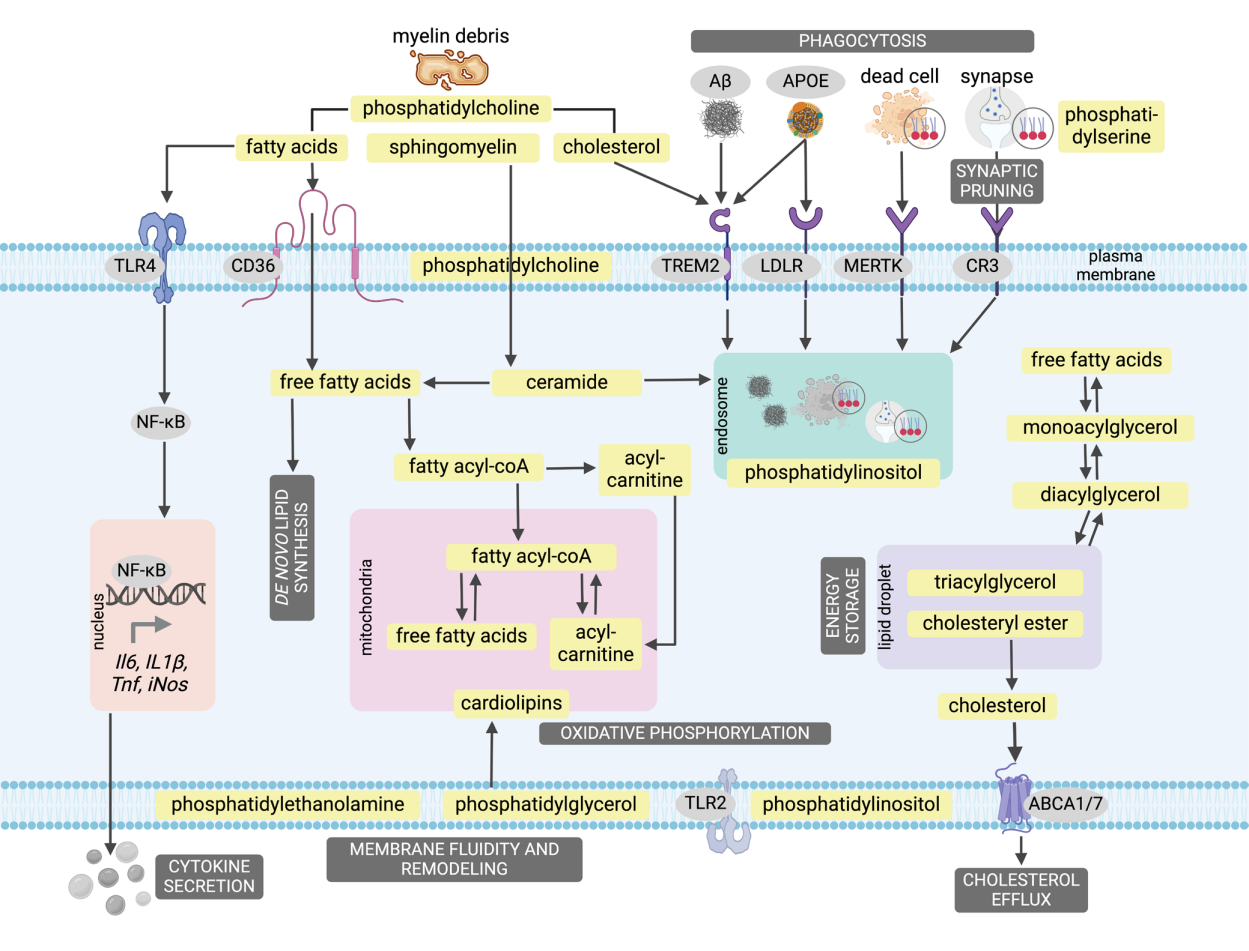
Major lipid pathways and regulation in microglia. Select pathways and mechanisms (dark gray) mediated by lipids (yellow) in microglia. The microglial lipidome can be altered by external triggers such as dying cells, lipoproteins, and misfolded proteins. Lipids from the extracellular space interact with key membrane proteins on the microglial cell surface and are subsequently metabolized within the cells. For instance, free fatty acids can be metabolized by fatty acyl‐CoA within mitochondria or stored in lipid droplets via the acylglycerol pathway. Several species of phosphatidylinositols (PIs) influence microglial endolysosomal function and the phagocytic clearance of debris. Cholesterol is transported out of the cell via ABCA1/7 transporters. Certain lipids also modulate the microglial inflammatory response through the TLR4 and NF‐kB pathway. Phosphatidylcholines, phosphatidylinositols, phosphatidylglycerols, and phosphatidylethanolamines embedded in the microglial cell membrane regulate membrane fluidity and remodeling, thereby influencing cellular integrity, migration, and response to stimuli. It is important to note that this is a broad and simplified overview of lipid‐mediated pathways in microglia. In reality, these molecules and pathways are far more complex, intertwined, and susceptible to changes even from subtle environmental shifts.

Lipids play a significant role in regulating microglial states and functions (Fahy et al. 2005). They are known to influence key microglial processes such as phagocytosis, migration, and energy production. The cellular lipidome exhibits remarkable structural diversity, comprising various lipid types categorized into distinct classes, each implicated in nearly all aspects of cellular functions (Figure 1). Lipids play essential roles in modulating membrane protein activity on the cell surface, thereby directly impacting microglial function. For instance, phospholipids, such as phosphatidylcholines (PCs), promote phagocytosis by acting as ligands for transmembrane proteins like Triggering receptor expressed on myeloid cells 2 (TREM2) (Nugent et al. 2020; Wang et al. 2015) and low‐density lipoprotein receptors (LDLRs) (Shi et al. 2021). Apoptotic cells expose phosphatidylserines (PSs) on their membranes that act as “eat me” signals and recruit microglia for phagocytic clearance (Scott‐Hewitt et al. 2020; Park et al. 2021). Additionally, lipids facilitate the incorporation of membrane‐spanning proteins within the lipid bilayer and directly signal to proteins during normal cellular functions. Intracellular lipids are also actively involved in several key processes, such as the tricarboxylic acid cycle and oxidative phosphorylation (Figure 2) (Yang et al. 2021). Thus, both the structural and signaling roles of lipids are integral to maintaining cellular state and function (Figure 2 and Table 1).

**TABLE 1:** Listing lipid classes related to microglial function, model system, and potential targets in health and disease.

Lipid class	Subclass/identity	Microglial function	Model system/context	Known or predicted target(s)	Citation
Phospholipids	Phosphatidylcholine	Injury response	Adolescent female mice, Spinal nerve injury		Xu et al. (2016)
	Oxidized phosphatidylcholines	↑Phagocytosis, damage mediation	Adolescent‐young adult female mice, Multiple sclerosis (EAE)	TREM2	Dong et al. (2021)
	Phosphatidylinositol 4,5‐bisphosphate	↑TLR2, TLR4 signaling	BV2 microglia, neonatal microglia	TIRAP	Nguyen et al. (2017)
	Phosphatidylserines	↑Phagocytosis, ↑Synaptic pruning	Neonatal microglia, P10, P18, and P30 mice, development	TREM2	Scott‐Hewitt et al. (2020)
			1–2‐month‐old mice, Seizure	MERTK	Park et al. (2021b)
			6‐month‐old mice, Alzheimer's disease	TREM2	Rueda‐Carrasco et al. (2022)
			P4,5,10,30 mice	GPCR56	Li et al. (2020)
	Phosphatidylglycerols	Unknown	Mouse microglia in vitro, aging		Fitzner et al. (2020)
Fatty acyls	Palmitic acid	↓Viability	BV2 microglia	CD36	Urso and Zhou (2021)
		↓Phagocytosis, ↓Migration, △Inflammation marker transcription	Neonatal rat microglia, IFN‐γ treatment	NOS2, IL1B, IL6, IL12B, MIP1A, H2AB, TNFA	Yanguas‐Casás et al. (2018)
	Long‐chain saturated free fatty acids	Acute inflammatory response	WT adult mouse microglia ex vivo, Alzheimer's disease, multiple sclerosis	ELOVL6	Wang et al. (2012); Garcia Corrales et al. (2023)
	Monounsaturated fatty acids	Inflammatory phagocytic phenotype	Mouse microglia	SCD1	Bogie et al. (2020)
	Unsaturated fatty acids (linoleic acid, oleic acid, docosahexaenoic acid)	↑Viability, ↑Neutral lipid accumulation	Palmitic acid treated BV2 microglia		Lancaster et al. (2018)
	Docosahexaenoic acid	↑ Aβ_42_ Phagocytosis	CHME3 microglia, Alzheimer's disease		Xu et al. (2016)
		↓ TLR4 signaling	BV2 microglia		Madore et al. (2020)
	Eicosapentaenoic acid	↑ Aβ_42_ Phagocytosis	CHME3 microglia, Alzheimer's disease		De Smedt‐Peyrusse et al. (2008)
	n‐3 poly unsaturated fatty acids	Synaptic pruning regulation	P21 mice, maternal diet		(Madore et al. 2020)
	L‐Carnitine	↓NO production	SIM‐A9 microglia		Gill et al. (2018)
	Acyl‐L‐Carnitine	↓CDllb staining density	8–9‐week‐old male mice, Parkinson's disease		Burks et al. (2019)
	Lipoxin A4	Pro‐resolving phenotype	CD1 mice, Wistar rats, Spinal cord injury	ALX/FPR2	Martini et al. (2016)
	Leukotriene B4, C4	Viral resistance	Human microglia‐like cells, HIV infection		Bertin et al. (2012)
	Prostaglandin E2	Sexual differentiation	P0‐65 rats, development		Lenz et al. (2013), Bordt, Ceasrine, and Bilbo (2020)
Sterol lipids	Cholesterol	Cholesterol clearance	Young adult, aged mice, aging	TREM2, APOE, ABCA1	Nugent et al. (2020), Berghoff et al. (2021), Loving and Bruce (2020), Feringa and van der Kant (2021)
		↓Remyelination, ↓Debris clearance	Young adult, aged mice, demyelinating disease	NLRP3 Inflammasome	Cantuti‐Castelvetri et al. (2018)
Glycerolipids	Triacylglycerols	↓Phagocytosis, ↑ROS production, ↑Pro‐inflammatory cytokine secretion, chronic inflammatory response	5–7‐month 5xFAD mouse and aged human AD, Lipid droplet accumulation	IL‐10, CCL3, CCL4, IL‐6, CCL5, TNF‐α, IL‐1β, IL‐1α, CXCL1 and CXCL10, DGAT2	Prakash et al. (2023), Marschallinger et al. (2020)

Despite the critical role of lipids, only a limited number of studies have explored how they drive microglial dysfunction under conditions of inflammation and disease. Dysregulated lipid metabolism in microglia contributes to neuroinflammatory processes and is implicated in neurodegenerative disorders such as Alzheimer's disease (AD) (Yin 2022), Parkinson's disease (PD) (Brekk et al. 2020), and Multiple Sclerosis (MS) (Dong et al. 2021). Identifying and characterizing specific lipids that influence microglial responses in disease could provide functional biomarkers, while genes and their protein products involved in lipid synthesis may offer new therapeutic targets (Table 2). Therefore, characterizing the functional lipidome that modulates microglial biology under normal and diseased conditions is essential for understanding how microglia contributes to disease‐associated disruption of tissue homeostasis. With the growing interest in microglial metabolism, this review provides a detailed overview of major lipid classes and their roles in regulating microglial biology. Additionally, we focus on lipid droplets (LDs), lipid‐rich organelles that influence microglial lipid metabolism and function (Table 3). Finally, we review the analytical tools and strategies for studying lipid dynamics in microglia and highlight open questions for future research on microglial lipid metabolism in both health and disease.

**TABLE 2:** Lipid metabolism and genes related to their function.

Function	*Gene* (NCBI Gene ID)
Monoacylglycerol metabolism	***MGLL*** (11343), ***MGAT1*** (4245)
Diacylglycerol metabolism	***PLCG1*** (5335), *PLPP1* (8611), *DGAT1* (8694), *DGAT2* (84649)
Triacylglycerol transport/metabolism	***APOC 2*** (344), ***APOE** (*348), ***C3*** (718), ***LIPA*** (3988), ***LPL*** (4023), ***DGAT1*** (8694), ***DGAT2*** (84649)
Cholesterol transport/metabolism	***ABCA1*** (19), ***ABCG1*** (9619), ***ACAT1*** (6646), ***CH25H*** (9023), ***DHCR7*** (1717), ***FDFT1*** (2222), ***FDPS*** (2224), ***HMGCR*** (3156), ***HMGCS1*** (3157), ***INSIG1*** (3638), ***LIPA*** (3988), ***LDLR*** (3949), ***MSMO1*** (6307), ***NCEH1*** (57552), ***NPC2*** (10577), ***NR1H3*** (10062), ***NSDHL*** (50814), ***PRKAB1*** (5564), ***SREBF2*** (6721), ***SC5D*** (6309)
Phospholipid transport/metabolism	***ABCA1*** (19), ***ABCG1*** (9619), ***LPCAT2*** (54947), ***MBOAT1*** (154141), ***PTEN*** (5728), ***PIK3CD*** (5293), ***PLA2G7*** (7941), ***PLA2G15*** (23659), ***PLCG1*** (5335), ***PLPP1*** (8611), ***SLC27A1*** (376497), ***TREM2*** (54209)
Fatty acid metabolism/transport	***ACACA*** (31), ***ACADS*** (35), ***CD36*** (948), ***DEGS1*** (8560), ***ELOVL1*** (64834), ***ELOVL2*** (54898), ***ELOVL3*** (83401), ***ELOVL4*** (6785), ***ELOVL5*** (60481), ***ELOVL6*** (79071), ***ELOVL7*** (79993), ***FABP3*** (2170), ***FASN*** (2194), ***MLYCD*** (23417), ***MGLL*** (11343), ***PRKAB1*** (5564), ***SLC27A1*** (376497), ***FADS1*** (3992), ***FADS2*** (9415)
Eicosanoid metabolism	***LTC4S*** (4056), ***PTGS1*** (5742), ***PTGS2*** (5743)
Undefined lipid metabolism/transport	***ANGPTL3*** (27329), ***APOC1*** (341), ***CH25H*** (9023), ***ECHS1*** (1892), ***NCEH1*** (57552), ***SLC27A1*** (376497)

**TABLE 3:** Select studies involving lipid droplet‐laden microglia in AD and other diseases.

Study	Identification of LD^+^ microglia in vivo	Species and region specificity	Induction of LD^+^ microglia in vitro	Function of LD^+^ microglia	Key target to modulate LD^+^ microglial function	Analytical methods for lipid/LD analysis
LD^+^ microglia in AD						
Prakash et al. (2023).	DGAT2^+^ LD^+^ microglia surrounding Aβ plaques	Human AD patients, 5xFAD mouse brains (subiculum, hippocampus)	Aβ‐induced primary mouse microglia ex vivo and in vitro	Reduced phagocytic capacity of Aβ	DGAT2	Fluorescence microscopy, flow cytometry, Raman spectroscopy, direct infusion multiple reaction monitoring (MRM)‐profiling, ion/ion charge inversion for fatty acid structural verification
Haney et al. (2024).	ACSL1^+^ LD^+^ microglia observed near Aβ plaques	Human AD patients with APOE4/4 genotype	Fibrillar Aβ induces ACSL1 expression in human iPSC‐derived microglia	LD^+^ microglia induce high concentrations of pTau and apoptosis in neurons	N/A	Fluorescence microscopy, Raman microscopy, electron microscopy, flow cytometry, liquid chromatography combined with mass spectrometry (LC–MS)
Li et al. (2024).	LD^+^ microglia near AT8^+^ neuronal processes	P301S tauopathy mice (hippocampus) and tau‐expressing fly brains	Transfer of unsaturated lipids from tauopathy iPSC neurons to BV2 cells	LD^+^ microglia are proinflammatory and exhibit reduced phagocytosis capacity	Neuronal AMPK	Electron microscopy, Fluorescence microscopy, Raman spectroscopy/imaging
Liang et al. (2024).	LD^+^ microglia increase in AD mice fed high fat diet	9‐month‐old 3xTg mice (hippocampus)	N/A	LD^+^ microglia increase in number and size in AD mice fed a high fat diet. Associated increases seen in pTau, Aβ, and T cell infiltration	N/A	Fluorescence microscopy, flow cytometry
LD^+^ microglia in other contexts (aging, stroke, injury)						
Victor et al. (2022).	N/A	Human female iPSCs, AG09173 & AG10788 (sporadic AD) edited to have APOE4/4 genotype	Oleic acid supplemented culture medium	LD^+^ microglia with APOE4/4 mutation were less responsive to ATP	ACSL1	Fluorescence microscopy
Wei et al. (2024).	LD^+^ microglia in hypoxic conditions	C57Bl/6J neonatal mice (cortex), male 10–12‐week‐old mice (cortex, striatum)	Oxygen–glucose deprivation and TREM2 knockdown	TREM2 knockdown reduced phagocytosis following oxygen–glucose deprivation	TREM2	Fluorescence microscopy, flow cytometry, fluorometric assay
Li et al. (2023)	LD^+^ microglia in the infarct core 3‐ and 7‐days post ischemic stroke	Mouse with middle cerebral artery occlusion surgery	Treatment of LPS and cellular debris on primary mouse microglia	LD^+^ microglia were mostly anti‐inflammatory, and reduction of LDs induced pro‐inflammatory phenotype	ATGL	Fluorescence microscopy, flow cytometry
Arbaizar‐Rovirosa et al. 2023).	LD^+^ microglia 24 h after ischemia (LD formation associated with type I interferon response)	Mouse with middle cerebral artery occlusion surgery	N/A	LD‐enriched microglia display impaired phagocytosis of pHrodo *E. coli* particles	N/A	Fluorescence microscopy, electron microscopy
Marschallinger et al. (2020).	LD^+^ microglia in the aged brain	Aged human brains (hippocampus)	LPS‐treated BV2 cells	LD^+^ microglia phagocytose significantly fewer A555^+^ myelin particles in vivo	GRN	Fluorescence microscopy, electron microscopy, direct infusion mass spectrometry
Byrns et al. (2024).	LD^+^ microglia in aged fly brain	40‐day‐old *Drosophila*	N/A	AP1^+^ microglia may be reservoirs of FFAs contributing to lipid accumulation in AP1^−^ microglia	AP1, SREBP	Fluorescence microscopy, direct infusion MRM‐profiling
Claes et al. (2021)	LD^+^ microglia‐like cells found near Aβ plaques	Human iPSCs (AICS‐0036) with TREM2‐R47H mutation transplanted into 5XFAD‐hCSF1 mice (hippocampus)	N/A	LD^+^ microglia‐like cells with AD protective TREM2 mutation have reduced LD area. Plaque proximal LD^+^ microglia‐like cells have a transcriptional signature similar to atherosclerotic foam cells.	TREM2	Fluorescence microscopy
Stephenson et al. (2024).	N/A	Human iPSC‐derived microglia (APOE3 and APOE4 microglia)	Treatment of LPS on human iPSC‐derived microglia	LPS‐induced LD^+^ microglia show increased phagocytosis of Zymozan particles, dextran, and Aβ	DGAT1/2, ACSL1, ATGL	Fluorescence microscopy
Nugent et al. (2020).	CE, oxCE, and TG‐enriched microglia in Trem2^−/−^ brain	Microglia from Trem2^−/−^ mice 12‐week after demyelination with CPZ treatment (forebrain)	Treatment with myelin	Microglia lacking TREM2 store esterified cholesterol from myelin in LDs	TREM2, ACAT1, APOE	Fluorescence microscopy, LC–MS MRM, surface plasmon resonance

## 2 Major Lipid Classes and Their Role in Microglial Biology

The cellular lipid landscape is vast and complex. The International Lipid Classification and Nomenclature Committee, under the sponsorship of the LIPID MAPS Consortium, has classified lipids into eight main categories, with each with its own subclassification hierarchy (Fahy et al. 2009, 2007). These eight primary classes of lipids are: fatty acyls, glycerolipids, glycerophospholipids (also referred to as phospholipids) (Fahy et al. 2009), sphingolipids, sterol lipids, prenol lipids, saccharolipids, and polyketides. Each class contains hundreds, if not thousands, of distinct “lipid species,” which differ based on factors such as acyl chain length, presence or absence of C=C bonds, the number and location of C=C bonds, and the functional groups attached to the lipid structures. This results in each lipid class containing unique subclasses of molecules and exhibiting extensive structural diversity (Figure 1). In this review, we examine several of these major lipid classes and their subclasses.

### 2.1 Phospholipids

Within the CNS, phospholipids originate from several sources, including endogenous synthesis, dietary consumption, and lipid remodeling pathways. Pathways such as the Kennedy pathway and mitochondrial lipid metabolism are primarily responsible for *de novo* phospholipid synthesis, while lipid remodeling and the interconversion of lipid precursors, often from dietary sources, maintain the diversity and balance of phospholipids required for various cellular and metabolic functions. Phospholipids are major components of cell membranes, influencing membrane fluidity and the functions of embedded membrane proteins (Divecha and Irvine 1995; Dai, Tang, and Pang 2021). In general, phospholipids consist of a central glycerol backbone, two fatty acyl chains, and a phosphate group substituted with one of the following polar head groups: inositol (PI), choline (PC), serine (PS), ethanolamine (PE), or glycerol (PG) (Figure 1). Other minor membrane phospholipids include sphingomyelin and cardiolipin (CL), the latter being a mitochondria‐specific phospholipid (Figure 2). Each subclass of phospholipids exhibits unique functions within and outside the cellular environment. Despite their seemingly simple building blocks, the structural diversity of phospholipids arises from variations in fatty acyl composition, head group identity, and fatty acid linkage types (Figure 1) (Yetukuri et al. 2008).

#### 2.1.1 Phosphatidylcholines (PCs)

PCs are the most abundant phospholipids, comprising 40%–50% of total phospholipids. The Kennedy pathway is primarily responsible for the synthesis of PCs within the CNS, utilizing choline from both dietary sources and systemic circulation. PCs also serve as a source of choline for the production of the neurotransmitter acetylcholine. In addition to being essential architectural components of plasma membranes, PCs play a major role in dictating membrane fluidity and dynamics (Figure 2). Consequently, PCs are key players in membrane‐mediated signaling events (van Meer, Voelker, and Feigenson 2008; Blank, Enzlein, and Hopf 2022). Studies in macrophages, as well as recent research with microglia, have shown that PC synthesis is crucial for the secretion of inflammatory cytokines (Tian et al. 2008; Okada et al. 2022). PCs also act as ligands for TREM2 on the microglial surface, initiating downstream signaling events (Wang et al. 2015; Sudom et al. 2018). Changes in PC levels are correlated with microglial reactivity during injury. One study demonstrated an increase in arachidonic acid‐containing PC (specifically [PC 16:0_20:4 + K]^+^) associated with reactive microglia in the spinal cord following peripheral nerve injury. This specific PC species could potentially serve as a marker to identify reactive microglia in spinal cord injury conditions.

Oxidized phospholipids, including oxidized PCs (OxPCs) contribute to increased inflammation in the CNS during disease. Exogenous OxPCs are considered potent neurotoxins and are found in MS lesions in the brain, where they drive neurodegeneration (Dong et al. 2021). Microglia can directly interact with these toxic OxPCs in a TREM2‐dependent manner, with TREM2^high^ microglia being required for OxPC elimination, potentially preventing further neuronal damage in MS (Dong et al. 2021). Microglia have been shown to protect neurons against OxPC toxicity by eliminating them from their microenvironment via phagocytosis. Notably, the loss of microglia exacerbated OxPC‐mediated neurodegeneration in vivo (Dong et al. 2021). In another study, OxPC‐induced neurodegeneration was exacerbated in the spinal cords of aged mice, with genes associated with lipid processing, such as *Trem2*, *Apoe*, *Lpl*, *Igf1*, *Abcg1*, and *Abca1* (Table 2), detected at higher levels in microglia associated with these OxPC lesions (Dong et al. 2022). This microglial transcriptional programming in response to OxPC‐mediated neurodegeneration was also identified in postmortem brain tissue sections from patients with MS (Dong et al. 2022). These findings further emphasize how specific PC lipid species regulate microglial state and function in disease‐specific environments.

#### 2.1.2 Phosphatidylinositols (PIs)

PIs are minor phospholipid components compared to their PC and phosphatidylethanolamine (PE) counterparts. The brain is particularly rich in PI lipids, where they can amount to ~10% of the phospholipid content. PI is synthesized from inositol and cytidine diphosphate‐diacylglycerol. PIs are important signaling lipids, acting as key regulators of signal transduction at neuronal synapses. Moreover, PI lipids serve as cellular membrane identifiers based on their location within the cell. In addition, PIs provide membrane charge and facilitate interactions with peripheral and integral membrane proteins. For example, phosphatidylinositol 4,5‐bisphosphate (PI(4,5)P_2_) marks the plasma membrane, phosphatidylinositol 3‐phosphate (PI(3)P) defines early endosomes, and phosphatidylinositol 3,5‐bisphosphate (PI(3,5)P_2_) marks late endosomes (Figure 2) (Wallroth and Haucke 2018). These PI lipids, along with PI(3,4,5)P_3_, PI(3,4)P_2_, and so on regulate microglial actin remodeling and phagocytosis (Desale and Chinnathambi 2021) (Figure 2). Under normal conditions, PIs (along with PSs) are restricted to the inner leaflet of the plasma membrane. During cellular stress or apoptosis, the membrane may “flip,” exposing PIs to the extracellular space, where they act as “eat me” signals for phagocytosis.

PIs also serve as second messengers acting as precursors to other signaling molecules (Olivença et al. 2018). Proteins that interact directly with PIs have been shown to regulate innate immune responses in microglia. For example, phosphatidylinositol 4‐phosphate 5‐kinase α (PIP5Kα), a protein involved in the synthesis of phosphatidylinositol 4,5‐bisphosphate (PIP_2_), can influence microglial response to pathogens by modulating the levels of toll‐like receptors (TLRs) on the cell membrane (Table 1). PIP_2_ is relatively abundant in the plasma membrane, where it plays a critical role in regulating physiological processes such as phagocytosis, receptor endocytosis, and lipid‐mediated cell signaling. In microglia, elevated PIP_2_ levels can activate signaling cascades downstream of TLR2 and TLR4. Knockdown of PIP5Kα reduces the pro‐inflammatory response in microglia, leading to decreased levels of interleukin 1β (IL‐1β), IL‐6, tumor necrosis factor (TNF), and inducible nitric oxide synthase (iNOS). Another study demonstrated that PIP_2_ recruits key adaptor proteins, such as myeloid differentiation factor 88 (MyD88) and Toll/IL‐1 receptor domain‐containing adaptor protein (TIRAP), to the cell membrane, facilitating TLR4 signaling in microglia. Thus, PIP_2_ positively regulates TLR‐mediated inflammatory responses in microglia by facilitating the recruitment of adaptor proteins like MyD88 and TIRAP to the plasma membrane (Nguyen et al. 2013).

A PI‐associated enzyme that has recently gained significant attention in AD research is inositol polyphosphate‐5‐phosphatase (INPP5D, also known as SHIP1). INPP5D hydrolyzes phosphatidylinositol‐3,4,5‐trisphosphate (PI(3,4,5)P_3_) to produce PI(3,4)P_2_. Elevated expression of *INPP5D* has been linked to an increased risk of developing AD and is highly enriched in plaque‐associated microglia in both mouse and human AD brains (Tsai et al. 2021; Castranio et al. 2023). Studies have shown that *Inpp5d* deficiency in microglia increases the number of plaque‐associated microglia *numbers* (Samuels et al. 2023). The positive correlation between INPP5D levels and amyloid‐associated microglia suggests that INPP5D could serve as a biomarker for microglia in AD. This finding underscores the need for improved tools and methods to identify lipids, such as PI(3,4)P_2_, as novel biomarkers in AD and related diseases. Mass spectrometry‐based lipid profiling holds great promise for these studies. For example, an unusual PI lipid, PI 20:4/20:4, was shown to increase by 300% following macrophage activation (Gil‐de‐Gómez et al. 2013). Whether this lipid is similarly elevated in microglia under pathological conditions remains unknown. These studies further support the idea that bioactive lipids could serve as cellular biomarkers to detect changes in microglial states in various pathological conditions.

#### 2.1.3 Phosphatidylserines (PSs)

PSs are synthesized endogenously from PE or PC precursors via base‐exchange reactions catalyzed by PS synthases (PSS1 and PSS2). PSs are typically confined to the inner leaflet of healthy cell membranes but are exposed on the outer membrane during cell death, with the help of phospholipid‐flippase chaperones such as CDC50A (also known as TMEM30A) (Li et al. 2021). These exposed PS molecules on dying cells serve as “eat me” signals, recruiting microglia for their phagocytic clearance (Segawa and Nagata 2015) (Figure 2). This process is commonly observed during brain development, where excess synapses are pruned to support the formation of activity‐dependent neuronal connections and plasticity (Scott‐Hewitt et al. 2020). Neuronal synapses marked for “eating” or pruning display high levels of PS on their outer membranes, signaling microglia for phagocytosis (Maruoka and Suzuki 2021). Aberrant PS expression can disrupt synaptic refinement, impair myelin clearance, and affect neuronal connectivity during brain development, potentially leading to neurodevelopmental disorders. The specific PS species that are involved in these processes remained undefined (Safaiyan et al. 2021).

Notably, microglial recognition of PS and the phagocytic uptake of apoptotic cells is receptor mediated (Maruoka and Suzuki 2021; Safaiyan et al. 2021). Microglial receptors, such as TREM2 (Rueda‐Carrasco et al. 2022), MER Proto‐Oncogene, Tyrosine Kinase (MERTK) (Park et al. 2021), and G protein‐coupled receptor 56 (GPR56) directly bind PS ligands and activate phagocytic signaling pathways, leading to the engulfment of dead cells (Figure 2) (Li et al. 2020) (Table 1). In cases where microglia become dysfunctional and fail to clear dying neurons or other cells, targeting PS molecules on cell membranes or modulating the PS‐receptor axis could offer a strategy to enhance cell clearance.

#### 2.1.4 Phosphatidylethanolamines (PEs)

PEs are the second‐most abundant class of membrane phospholipids, comprising 25% of the total phospholipids in cell membranes, and are synthesized endogenously via two independent pathways (Vance 2008). First, in the endoplasmic reticulum (ER), PE is made via the Kennedy pathway. Second, in the mitochondria, PS is converted into PE by the mitochondrial enzyme PS decarboxylase. PEs are known to increase the membrane curvature and fluidity, facilitate synaptic vesicle formation, and promote membrane fusion within the CNS (Germain, Iraji, and Bakovic 2023). Though the major phospholipid structure consists of two esterified fatty acyl chains, plasmalogen PEs are characterized by the presence of an alkenyl chain attached to the *sn*‐1 position of the glycerol backbone and an ester chain attached to the *sn*‐2 position. In neuronal membranes, plasmalogen PEs can account for up to 90% of total PE lipid content (Braverman and Moser 2012), highlighting their crucial roles in neuronal function. In addition to being structural constituents of neuronal membranes, plasmalogen PEs serve as sources of arachidonic and docosahexaenoic acids and are implicated in protection against oxidative stress. Limited research has been conducted on how changes in PE levels and metabolism influence microglial states and functions, presenting a significant opportunity for further investigation. Insights from studies on peripheral immune cells can help identify PE‐related pathways that warrant closer examination in microglia. For example, in the liver, PEs are involved in the secretion of lipoproteins (van der Veen et al. 2017), though their role in microglial production of apolipoproteins in the brain, such as apolipoprotein E (APOE) and apolipoprotein J (APOEJ), remains unclear. Given their abundance in the cell membranes and their close association with PS metabolism (Vance 2008), more studies are needed to explore how microglial PEs influence phagocytosis and other cellular functions.

#### 2.1.5 Phosphatidylglycerols (PGs)

PGs are key components of the cell membrane and serve as intermediates in the synthesis of CLs, which are located in the inner mitochondrial membrane and play a role in oxidative phosphorylation (Figure 2). Despite their importance in maintaining membrane fluidity, stability, and supporting the respiratory electron transport chain, little is known about them in the context of microglia. Several processes in the periphery have defined the role of PGs. In macrophages, PGs regulate innate immune responses by inhibiting inflammation triggered by the bacterial endotoxin lipopolysaccharide (LPS), specifically by blocking the activation of TLR2 and TLR4 receptors (Choudhary et al. 2019). Additionally, in peripheral tissues, PGs have been shown to interfere with the binding of virus particles to cell membrane receptors, preventing viral uptake into host cells (Table 1). In mildly stressed and aged yeast cells, PG levels increase in mitochondria by 1.2‐fold, likely as intermediates for the rapid synthesis of CLs to combat oxidative stress. Whether these mechanisms are conserved in microglia remains to be investigated. Lipidomic analysis of cultured primary microglia revealed that, under physiological conditions, microglia are enriched with high concentrations of PGs (Fitzner et al. 2020). However, the functional significance of this elevated PG content in microglia under basal conditions is still unclear. However, the functional significance of this elevated PG content in microglia under basal conditions is still unclear. Further research is needed to determine how the roles of PGs in other cell types, including peripheral macrophages, can be translated to microglial function in the CNS.

### 2.2 Fatty Acyls

Representing one of the eight classifications of lipids, fatty acyls are a ubiquitous and diverse group of lipid molecules biosynthesized by the chain‐elongation of acetyl‐CoA (Figure 1). All lipid species within the fatty acyl class share the same molecular structure—they comprise a hydrocarbon chain with a carboxyl group. Given that the carboxyl group can be linked to a diverse array of substituents, the fatty acyl class encompasses tens of thousands of lipid species. This structural diversity is further enhanced by variations in the hydrocarbon chain, including differences in acyl chain lengths, the presence of branching or linear structures, and variations in the number, position, and geometry of unsaturation sites.

They are best known for serving as key architectural components of complex lipids, including, but not limited to, triacylglycerides, glycerophospholipids, and cholesteryl esters. While fatty acyls are most commonly found in their esterified form as part of complex lipid structures, they can also exist as free circulating molecules, referred to as free fatty acids (FFAs). Notable subclasses of biologically relevant fatty acyls include signaling molecules like eicosanoids and transport molecules such as acylcarnitines (ACs).

#### 2.2.1 Fatty Acids

Fatty acids are a prominent subclass of fatty acyls. In general, fatty acids are categorized as carboxylic acids containing either a saturated hydrocarbon chain (i.e., no carbon–carbon double bonds) or an unsaturated hydrocarbon chain (i.e., at least one carbon–carbon double bond) (Figure 1). Modifications to the hydrocarbon chain, including nitrosylation, cyclopropanation, and methyl chain branching, can occur, impacting the biochemical and biophysical properties of cellular membranes. While fatty acids are most often found in an esterified form as a building block of complex lipids, they can also exist as free circulating molecules following the lipolysis of triacylglycerides. The presence of excess fatty acids in their non‐esterified form (i.e., FFAs) is considered indicative of dysregulated lipid homeostasis (Eckel, Grundy, and Zimmet 2005).

FFAs undergo oxidative degradation via β‐oxidation in mitochondria and peroxisomes, which is essential to prevent FFA‐induced toxicity (Schönfeld and Reiser 2017). Additionally, long‐chain and very long‐chain saturated FFAs (with 14 and 20 plus carbon atoms, respectively) can induce cellular dysfunction and lipotoxicity (Engin 2017). For example, excess palmitic acid, a saturated FFA containing an aliphatic chain with 16 carbon atoms (C16:0), has been shown to promote inflammation and toxicity in microglial cells both in vitro and in vivo (Urso and Zhou 2021) (Table 1). Palmitic acid is metabolized into phospholipids, ceramides, or diacylglycerols; however, during chronic stress or injury, disruptions in these metabolic pathways can lead to an accumulation of these lipids. This buildup may cause ER stress and increase the production of reactive oxygen species in microglia (Urso and Zhou 2021; Yu et al. 2024). Palmitic acid can also impair the protective responses of microglia by affecting their phagocytic ability and migratory activity. Specifically, palmitic acid has been shown to suppress microglial phagocytic capacity and migratory ability under Interferon‐gamma (IFN‐γ)‐induced inflammatory condition.

Other relevant saturated FFAs implicated in microglia‐mediated inflammation include stearic acid (C18:0) and arachidic acid (C20:0). Treatment of microglial cells with stearic acid has been shown to increase the expression of pro‐inflammatory cytokines, such as TNF, IL‐1B, and IL‐6, via the nuclear factor‐kappa B (NF‐kB) signaling pathway (Wang et al. 2012). Furthermore, long and very‐long chain saturated FFAs (C16:0–C36:0) secreted by neurotoxic reactive astrocytes have been shown to cause toxicity in neurons and oligodendrocytes, but not in microglia (Guttenplan et al. 2021). Understanding how microglia withstand this lipid‐induced cytotoxicity under chronic inflammatory conditions may provide insights into their capacity for self‐protection, potentially by transitioning into a different cell state; this remains an open area of investigation. Microglia can eliminate excess long and very‐long chain FFAs through β‐oxidative degradation. They can also sequester these FFAs into lipid droplets (LDs) by converting them into triacylglycerols (TGs) (Figure 2). In fact, using mass spectrometry‐based lipidomics, we recently showed that microglia upregulate certain long‐chain FFAs (C19:0, C20:0, and C22:0) after acute (1‐h) Aβ treatment (Prakash et al. 2023). Twenty‐four hours following Aβ exposure, the levels of these FFAs were reduced, while TG levels were significantly upregulated (Prakash et al. 2023). This suggests that microglia metabolize FFAs into TGs as part of their response to Aβ‐induced inflammation.

While saturated FFAs, such as palmitic acid and stearic acid, are primarily associated with pro‐inflammatory or neurotoxic cellular phenotypes (Wang et al. 2012), certain unsaturated FFAs, including mono‐ and polyunsaturated fatty acids (PUFAs) like oleic acid, arachidonic acid, and docosahexaenoic acid (DHA), are linked to both anti‐inflammatory/neuroprotective and pro‐inflammatory/neurotoxic phenotypes (Urso and Zhou 2021; Hjorth et al. 2013; De Smedt‐Peyrusse et al. 2008; Madore et al. 2020) (Table 1). In the APP^NL‐GF^ mouse model of AD, microglia were found to contain increased levels of several PUFAs, including arachidonic acid (C20:4), docosatetraenoic acid (C22:4), and docosahexaenoic acid (DHA; C22:6) (Lin et al. 2024). Targeting the enzyme lysophospholipid acyltransferase (LPCAT3), which is involved in the synthesis of C20:4, through genetic deletion of *Lpcat3* in microglia, led to reduced oxidative stress and inflammatory responses, enhanced Aβ plaque phagocytosis, and mitigated Aβ‐associated neuropathology (Lin et al. 2024). In another study, DHA was found to inhibit pro‐inflammatory cytokine production by preventing NF‐κB translocation to the nucleus (Pettit et al. 2013; Komatsu et al. 2003). Oleic acid has been shown to counteract the pro‐inflammatory phenotype by suppressing the phosphorylation of p65 and c‐Jun—key components of the NF‐κB signaling pathway—thereby exhibiting anti‐inflammatory effects in microglial cultures (Beaulieu et al. 2021). Thus, microglial responses to specific saturated and unsaturated FFAs are likely context‐dependent.

Further research is needed to determine how saturated and unsaturated FFAs influence microglial phenotypes and functions, and how these changes contribute to inflammation and disease progression. Targeted mass spectrometry analysis of FFA species in microglia across different disease contexts is a promising approach to identify key FFAs involved in cell metabolism under various conditions. Additionally, genetically targeting genes responsible for the production of these lipid species in rodent models (e.g., *Elovl1*, *Elovl3*, and *Elovl7* for very long‐chain saturated fatty acids, and *Elovl2* and *Elovl5* for polyunsaturated fatty acids) (see summary of genes related to lipid metabolism in Table 2) will be valuable for studying their role in regulating microglial states and functions within specific disease paradigms.

#### 2.2.2 Acylcarnitines (ACs)

ACs are a subclass of fatty acyls consisting of a fatty acyl chain esterified to a carnitine backbone (Figure 1) and play multiple vital roles within the CNS, including energy metabolism, cellular signaling, and neuroprotection. Carnitines, which are amino acid derivatives, are crucial for fatty acid metabolism and energy production, as they facilitate the transport of fatty acids across the mitochondrial membranes by combining with fatty acids to form ACs. Once inside the mitochondria, ACs are oxidized for energy production (Figure 2). In the plasma, ACs are degraded by esterases, aiding in the removal of metabolized products from cells (Dambrova et al. 2022). Studies investigating the roles of carnitines and ACs in microglia are largely limited to in vitro models. For example, treatment with L‐carnitine has been shown to inhibit LPS‐induced nitric oxide production in cultured microglia (Gill et al. 2018), demonstrating the role of L‐carnitine in modulating inflammation pathways in microglia. In another study, acetylated L‐carnitines were shown to reduce microglial reactivity in the MPTP (1‐methyl‐4‐phenyl‐1,2,3,6‐tetrahydropyridine) mouse model of PD (Burks et al. 2019). Specifically, treatment of acetylated L‐carnitines in these PD mice prevented dopamine loss, preserved tyrosine hydroxylase‐positive neurons, and reduced astrocyte reactivity, as indicated by lower glial fibrillary acidic protein levels (Table 1). However, how these neuronal and astrocytic changes influence microglia and their response to these lipids in this model remains unclear. Given the role of ACs in mitochondrial function, further investigations are needed to elucidate how they modulate mitochondrial mechanisms in microglia, including cell stress, calcium homeostasis, senescence, innate immune response, and energy metabolism in various pathologies.

#### 2.2.3 Eicosanoids

Eicosanoids are derived from the oxidation of polyunsaturated fatty acid precursors and play distinct roles in innate immune responses. They are broadly classified into three main categories based on their structure and biosynthetic pathways: prostaglandins (PGs), leukotrienes, and thromboxanes. Eicosanoids are typically synthesized from arachidonic acid precursors, dihomo‐γ‐linolenic acid, or eicosapentaenoic acid (Calder 2020; Harizi, Corcuff, and Gualde 2008). In the immune system, eicosanoids like lipoxins, resolvins, protectins, leukotrienes, thromboxanes, and PGs regulate inflammatory mechanisms. Lipoxins, resolvins, and protectins are typically anti‐inflammatory and pro‐resolving. For instance, lipoxin A4 (LXA4) plays important roles in recovery after injury (Martini et al. 2016) (Table 1). In a mouse model of spinal cord injury, LXA4 has been shown to reduce pain response behavior and microglial reactivity (reduced IBA1 levels) at the site of hemisection (Martini et al. 2016). Additionally, in the APP mouse model of AD, LXA4 treatment was associated with an increased ratio of IBA1 to Aβ levels, indicating a shift toward a pro‐resolving phenotype in microglia and improved cognitive function (Medeiros et al. 2013). Conversely, leukotrienes, thromboxanes, and PGs are considered pro‐inflammatory (Zhao et al. 2019). For example, in vitro studies using human microglia‐like cells showed that exposure to leukotriene B4 or leukotriene C4, following the addition of human immunodeficiency virus (HIV), reduced the susceptibility of these cells to infection (Bertin et al. 2012). These studies suggest that leukotrienes may potentially affect the microglial response to pathogens, potentially enhancing their ability to resist viral infections such as HIV.

PGs are eicosanoids derived from arachidonic acid, a 20‐carbon unsaturated fatty acid, and contain a five‐membered ring spanning carbons 8–12 (Smyth et al. 2009). Each PG is named with the prefix “PG,” followed by a letter (A–K) based on the type and position of the substituents on the ring, and a numerical subscript (1–3) indicating the number of double bonds in the alkyl substituents. Among all PGs, PGE_2_ is the most studied in the context of inflammation and immune responses. PGE_2_ is produced from arachidonic acid through a series of reactions involving the enzyme cyclooxygenase 2, which is the target of nonsteroidal anti‐inflammatory drugs such as ibuprofen and aspirin (Minami et al. 2001). Four PGE_2_ receptors have been identified, namely, EP1, EP2, EP3, and EP4 (Hata and Breyer 2004).

PGs are classically recognized for their roles in pain signal propagation and local inflammatory signaling (Kalinski 2012). PGE_2_ exhibits both pro‐inflammatory and anti‐inflammatory effects, depending on the inflammatory stimulus and the expression of its receptors on different cell types. For example, in vitro studies have shown that reactive microglia secrete PGE_2_, which induces rapid proliferation of astrocytes (Zhang et al. 2009), typically post‐mitotic under normal conditions. Uncontrolled astrocyte proliferation may become pathological, potentially leading to astroglioma, although further investigation is needed in vivo.

PGE_2_ has also been shown to be critical for microglia during the process of sexual differentiation in development. In rodents, the preoptic area (POA) in the hypothalamus is much larger in males with greater dendritic density and higher variability of astrocytic morphology. This sexual dimorphism is associated with reproductive behaviors seen in males. Masculinization of the POA is carried out by microglia and depends on the production of PGE_2_. In fact, artificially increasing the levels of PGE_2_ in the POA of neonatal females results in the display of male reproductive behaviors during adolescence (Lenz et al. 2013; Bordt, Ceasrine, and Bilbo 2020).

Additionally, EP2 receptor signaling in microglia has been shown to contribute to their dysfunction in AD, particularly impairing the phagocytosis of Aβ42 oligomers (Johansson et al. 2014). Deleting the EP2 receptor specifically in microglia restored their chemotaxis toward amyloid plaques and enhanced Aβ clearance in the APP‐PS1 mouse model of AD (Johansson et al. 2014). This EP2 deletion also improved spatial memory and cognitive function in these mice, highlighting the crucial role of the PGE_2_‐EP2 signaling axis in microglial function in AD.

The EP4 receptor in microglia is also implicated in increased susceptibility to diet‐induced obesity. PGE_2_ levels rise in the mediobasal hypothalamus during high‐fat diet feeding (Lee et al. 2021), and deleting EP4 in microglia significantly reduced weight gain and food intake, resulting in a “lean” phenotype (Niraula et al. 2022). Mechanistically, EP4 deletion reduced microglial internalization of pro‐opiomelanocortin neuronal processes in the hypothalamus, which regulate food intake and energy expenditure (Niraula et al. 2022). Together, these studies underscore the critical roles that PGs, particularly PGE2, play in regulating microglial function across various physiological and pathological contexts.

### 2.3 Sphingolipids

Sphingolipids, appreciated as valuable membrane components, constitute a lipid family characterized by the presence of a sphingoid base (Figure 1). This class includes lipids from the ceramide, sphingomyelin, and glycosphingolipid families. While they were originally thought to serve only structural functions, sphingolipids are now known to participate in a variety of cellular processes. For example, sphingolipids modulate cell migration, growth, apoptosis, cell–cell interactions, and inflammatory responses (Hannun and Obeid 2018). Importantly, they are crucial for the formation and maintenance of the myelin sheath within the CNS, underscoring their significance in neurological health and disease.

#### 2.3.1 Ceramides

Ceramides, often referred to as “stress lipids” due to their involvement in signaling pathways that regulate apoptosis, particularly in response to oxidative stress, neuroinflammation, or other cellular stressors in the CNS, are a subclass of sphingolipids composed of a sphingosine and a fatty acyl chain (Figure 1). The primary de novo synthesis pathway for ceramide formation involves a series of enzymatic reactions that convert simpler molecular precursors into ceramide lipids. Ceramides can also be formed in the CNS by the breakdown of other sphingolipid structures, such as sphingomyelin and glycosphingolipids.

Ceramides are well known as integral building blocks of sphingomyelin (Nikolova‐Karakashian and Rozenova 2010), a major component of the lipid bilayer. In addition to their structural roles in cellular membrane dynamics, ceramides also act as signaling molecules. For example, ceramides regulate cellular stress response pathways following exposure to LPS, TNFα, IL‐1β, irradiation, and among others (Halasiddappa et al. 2013). Ceramides have been shown to enhance microglial secretion of brain‐derived neurotrophic factor, which is essential for neuronal survival and growth (Nakajima et al. 2002). Exogenous short‐chain ceramides can also inhibit the secretion of pro‐inflammatory cytokines and reactive oxygen species like iNOS in LPS‐activated primary microglia (Jung et al. 2013). Furthermore, these short‐chain ceramides (C2–C8 acyl chain lengths) have been shown to inhibit TLR4 signaling in microglia, revealing their anti‐inflammatory and antioxidant effects (Jung et al. 2013).

Further studies are needed to evaluate the differences between short and long‐chain ceramides on microglial physiology and activation state. Research in macrophages has shown that ceramides are enriched in mature phagosomes during phagocytosis through increased activity of ceramide synthase (specifically CerS2) (Pathak et al. 2018). Since excess ceramides can be toxic to cells and may activate apoptotic pathways, macrophages can metabolize ceramides into acyl ceramides, which are stored in LDs as a means to render them biologically inactive and make the cells resistant to ceramide‐induced toxicity (Senkal et al. 2017). Whether microglia also utilize these mechanisms to evade ceramide‐induced lipotoxicity in specific disease‐context is yet to be determined.

### 2.4 Sterol Lipids

Sterol lipids are isoprenoid‐derived lipids present in nearly all living organisms. For example, sterols such as cholesterol and its derivatives have been found in a wide range of organisms, including mammals, plants, fungi, and bacteria. The general structure of sterol lipids includes three primary components: a steroid nucleus, a hydroxyl group, and a hydrocarbon side chain. Variations in the composition of the hydrocarbon side chain, linked at the D ring of the steroid nucleus, contribute to a diverse array of sterol lipid structures. Prominent examples of sterol lipids include free cholesterol, steroid hormones, cholesterol esters (CEs), and bile acids. Sterol lipids are well‐known for their ability to modulate membrane dynamics and are primarily recognized for their role in maintaining membrane structure.

#### 2.4.1 Cholesterol and Derivatives

Cholesterol, composed of a central fused hydrocarbon ring core, a flexible hydrocarbon tail, and a single polar hydroxyl group, is the most common and well‐studied steroid. The CNS contains the highest amount of cholesterol in the body—approximately 23%—all of which is synthesized locally since the blood–brain barrier prevents the entry of cholesterol‐containing lipoproteins into the brain (Dietschy and Turley 2004; Mahley 2016). Cholesterol is predominantly produced by astrocytes and mature oligodendrocytes, and its secreted form is transported into the extracellular space with the help of APOE/J chaperone proteins (Mahley 2016; Berghoff et al. 2021). It is rare to find free‐floating cholesterol within cells, as it is mainly present in its esterified form as CEs. The esterification of cholesterol is catalyzed by acyl CoA:cholesterol acyltransferase 1 (ACAT1), and the breakdown of CEs releases fatty acids and free cholesterol, which are stored in LDs along with other neutral lipids such as TGs (Berghoff et al. 2021; Gouna et al. 2021).

Microglia require high concentrations of astrocyte‐derived cholesterol for their survival, both in vitro and in vivo (Bohlen et al. 2017). They express lower levels of cholesterol‐biosynthesis genes compared to astrocytes and matured oligodendrocytes, making them deficient in autonomously producing cholesterol. As a result, they depend on other CNS cells in vivo or direct cholesterol supplementation in vitro for their survival (Berghoff et al. 2021; Gouna et al. 2021; Bohlen et al. 2017). Although microglia exhibit relatively lower levels of cholesterol biosynthesis under physiological conditions (Zhang et al. 2014), they are essential for maintaining cholesterol homeostasis in the CNS. This includes (i) the phagocytic clearance of cholesterol released from myelin during demyelination and normal aging (Nugent et al. 2020), (ii) cholesterol transport across cell membranes (Abe‐Dohmae et al. 2004; Loving and Bruce 2020) via ATP‐binding cassette (ABC) transporters like ABCA7, LDLRs, TREM2, and TLR4 (Nugent et al. 2020; Feringa and van der Kant 2021) (Figure 2), (iii) cholesterol metabolism within cells, and (iv) storage within LDs (Ikonen 2008).

Cholesterol bound to APOE lipoparticles is phagocytosed via the microglial TREM2 receptor, and TREM2‐deficit microglia fail to perform lipid hydrolysis and cholesterol transport (Nugent et al. 2020) (Table 1). Studies have reported defective microglial lipid metabolism in aged brains, where microglia are unable to metabolize CEs from myelin debris, leading to the formation of visible intracellular cholesterol crystals in these cells (Cantuti‐Castelvetri et al. 2018). The accumulation of cholesterol crystals can damage microglial lysosomes and can trigger severe inflammatory events via the activation of the inflammasome pathway (Cantuti‐Castelvetri et al. 2018). In conclusion, these studies highlight the critical roles of microglia in cholesterol sensing, clearance, transport, and metabolism in the CNS.

### 2.5 Glycerolipids

Membrane development, energy storage, and crucial intracellular signaling activities all depend on a diverse collection of glycerolipids. Glycerolipids are formed through the esterification of long‐chain hydrocarbon groups at the hydroxyl moieties of a central glycerol backbone. They can exist as mono‐, di‐, or tri‐substituted glycerols. The most well‐known glycerolipid is TG, also known as triglyceride. Variations in hydrocarbon chain composition and stereochemistry result in a vast number of potential glycerolipid structures, including enantiomers and isomers, contributing to the extensive structural heterogeneity within the glycerolipid class.

#### 2.5.1 Triacylglycerides (TGs)

TGs are neutral lipids comprising three fatty acids with an ester linkage to a glycerol backbone (Figure 1). They can be synthesized by two major pathways: the glycerol phosphate pathway and the monoacylglycerol pathway, both of which utilize fatty acyl‐CoAs as acyl donors. Notably, diacylglycerol acyltransferase (DGAT1/DGAT2) catalyzes the final and only committed step in TG biosynthesis, making these enzymes significant targets for modulating TG levels within the cell (Prakash et al. 2023; Yen et al. 2008) (Table 2). TGs are stored in the core of cytoplasmic LDs, which are enclosed by a phospholipid monolayer and associated with hydrophobic proteins such as perilipins. LDs are distinctive cellular organelles that serve as the primary fat/energy storage components and possess their own metabolic pathways and enzymes (Figure 2). LDs, which contain TGs and other lipids such as diacylglycerols, cholesterols (as cholesteryl esters), oxylipins, retinol esters, and coenzyme A esters, are key sites for lipid metabolism within cells. Since FFAs are sequestered to form TGs via DGAT1/2, TGs are considered reservoirs of fatty acids that can be hydrolyzed as needed by cells for use as structural components or substrates for various lipid metabolism pathways.

Although TGs are essential for the normal physiological function of cells, excess accumulation of TGs can indicate a dysregulated microglial state and function (Jarc and Petan 2020) (Table 1). For example, TG‐rich, LD‐accumulating microglia have been observed in aged mouse and human brains, displaying phagocytic defects, increased reactive oxygen species, and elevated proinflammatory cytokine profiles (Marschallinger et al. 2020). In addition to lipids, changes to the TG‐associated proteins like perilipins, long‐chain acyl‐CoA synthetases, and glycerol‐3‐phosphate acyltransferases contribute to differential lipid regulation (Coleman 2019) and can serve as targets for modulating lipid pathways in microglia.

TGs can also be secreted into the extracellular space and transported between cells with the help of very low‐density lipoprotein molecules, low‐density lipoprotein receptor‐related protein 1 (LRP1), and heparan sulfate proteoglycans (Loving and Bruce 2020; Loving et al. 2021) (Figure 2). Aberrant TG transport between cells could be a result of mutations in lipoprotein genes like *Apo*e and *Clu*—which are linked to the onset and progression of AD. In fact, microglia harboring the AD‐linked *APOE4* genotype show increased levels of TG‐rich LDs (Stephenson et al. 2024). Inhibiting TG biosynthesis in *APOE4* microglia attenuated the disease‐associated transcriptional state of the cell, indicating a close relationship between microglial TG levels and their immune state in AD (Stephenson et al. 2024). Taken together, these studies demonstrate that TGs are key immunomodulating molecules in microglia, not only in normal physiology but also in pathological conditions.

#### 2.5.2 Lipid Droplet‐Laden Microglia in Health and Disease

Although the functional consequences of LD accumulation in microglia have not been fully characterized, recent studies, including our own, have identified key roles of LD‐enriched (LD^+^) microglia in AD and other conditions such as stroke, traumatic brain injury, and aging (Prakash et al. 2023; Marschallinger et al. 2020; Haney et al. 2024; Li et al. 2024) (see Table 3 for a summary of selected studies investigating LD^+^ microglia in different diseases). We recently identified a plaque‐associated LD^+^ subtype of microglia in the brains of human AD patients and 5xFAD mice (Prakash et al. 2023). We found that exposure to Aβ is sufficient to induce LD formation in microglia, and that LD accumulation requires a specific metabolic conversion of FFAs to TGs via the DGAT2 enzymatic pathway (Prakash et al. 2023). The chronic accumulation of LDs resulted in deficits in microglial ability to clear Aβ via phagocytosis. Thus, we hypothesize that in chronic AD, microglia likely transition from “emergency responders” to “damage control” mode. Initially, they are active phagocytes, rapidly clearing Aβ during the early stages of disease onset. However, as the Aβ burden increases, microglia reduce their phagocytic activity and adopt a self‐protective state to cope with cellular toxicity. This shift in cell state is marked by changes in FFA levels and their conversion into TGs within LDs for long‐term energy storage. By reducing cellular LD levels via DGAT2 inhibition, we were able to improve microglial phagocytic ability, resulting in reduced plaque load *in vivo* (Prakash et al. 2023). To our knowledge, this work provides the first evidence of a lipid‐related neuroimmunotherapy for AD.

In a related study, human induced pluripotent stem‐cell (iPSC)‐derived microglia from APOE4/4 carriers, but not APOE3/3 carriers, accumulated LDs when treated with Aβ fibrils (Haney et al. 2024) (Table 3). Microglia from APOE4/4 human AD patients also contained more LDs, and these LD^+^ microglia were enriched for long‐chain fatty acyl‐CoA synthetase 1 (ACSL1), another enzyme in the TG pathway (Table 2). These ACSL1^+^LD^+^ microglia were toxic to neurons, leading to increased apoptosis and hyperphosphorylated tau production (Haney et al. 2024).

Besides Aβ, other pathological triggers can also induce LD accumulation in microglia (Table 3). In tauopathy models, neurons can transfer lipids to microglia, where they form LDs and trigger inflammation (Li et al. 2024). The pathological LDs generated by this process are regulated by neuronal AMP‐activated protein kinase and are conserved across mouse, fly, and cell culture models of tauopathy (Li et al. 2024). Functionally, these neuron‐induced LD^+^ microglia exhibited an increased oxidative stress and proinflammatory cytokine production, along with a reduction in phagocytic capacity (Li et al. 2024). In flies, neurons can transfer peroxidated lipids to glia via apolipoproteins, where they are stored as LDs (Haynes et al. 2024; Goodman, Moulton, and Bellen 2024), highlighting how neuronal activity can promote LD formation in glia. Interestingly, several genes and proteins required to facilitate the transfer of lipids from neurons to glia have also been implicated in AD. For example, genes encoding for APOE and APOJ lipoproteins, neuronal ABCA transporters and receptor‐mediated endocytic proteins like LRP1, phosphatidylinositol binding clathrin assembly protein (PICALM), CD2‐associated protein (CD2AP), among others, have been identified as AD risk factors (Victor et al. 2022; Moulton et al. 2021) (Table 2).

Microglial accumulation of LDs has been reported in the context of aging. LD^+^ microglia in aged brains exhibit a reduced phagocytic capacity and a pro‐inflammatory cell phenotype (Marschallinger et al. 2020) (Table 3). In aged fly brains, senescent glia responding to neuronal mitochondrial dysfunction can promote LD formation in non‐senescent glia via activator protein 1 (AP1) (Byrns et al. 2024) (Table 3). Targeting AP1 activity prevented LD accumulation in glia and increased the lifespan of the fly, demonstrating how mitochondrial function in neurons can influence LD formation and overall physiology in glia (Byrns et al. 2024).

LDs can also accumulate in microglia following acute ischemic injury (Arbaizar‐Rovirosa et al. 2023) (Table 3). In one study, LD^+^ microglia were detected at lesion sites in mouse brains as early as 3 days after middle cerebral artery occlusion stroke (Li et al. 2023) (Table 3). When adipose triglyceride lipase (ATGL), the enzyme that breaks down TGs into FFAs, was inhibited, microglial secretion of proinflammatory cytokines was significantly reduced. Furthermore, the mice exhibited decreased infarct volume and improved neurobehavioral performance during the acute stage of stroke (Li et al. 2023), correlating microglial LD changes with behavioral outcomes. The inflammatory response of LD^+^ microglia in stroke and injury may also involve TREM2. In another study, TREM2‐deficient microglia showed impaired phagocytosis and elevated CE levels, which led to LD formation and upregulation of the LD‐associated protein perilipin‐2 (Wei et al. 2024) (Table 3). Additionally, after ischemia in these TREM2‐knockdown mice, the LD^+^ microglia adopted a pro‐inflammatory phenotype, which was associated with increased neuronal damage and death (Wei et al. 2024). These studies collectively highlight the role of LDs in normal aging, as well as in injury and disease contexts, demonstrating that LDs modulate microglial functions in a context‐dependent manner in response to different pathological insults.

Astrocytes can also regulate LD metabolism in microglia. For example, the loss of cellular communication network factor 1 (CCN1) in white matter astrocytes after spinal cord injury resulted in aberrant activation of local microglia (McCallum et al. 2024). These lesion‐remote microglia, induced by astrocyte‐secreted CCN1, exhibited increased LDs and reduced phagocytic capacity to clear myelin debris following injury (McCallum et al. 2024). Future studies should explore the mechanisms of LD accumulation and cell function through glia–neuron crosstalk, including interactions with astrocytes, oligodendrocytes, ependymal cells, and microglia.

While there is evidence that microglial LD accumulation may be detrimental, there is also evidence suggesting a protective role for LDs in microglia. As mentioned earlier, LD accumulation reduces phagocytic capacity in 5XFAD microglia, and reduced lipid uptake in APOE4 microglia may lead to hyperpolarization of neurons due to increased extracellular cholesterol accumulation (Victor et al. 2022). On the other hand, it has been shown that LD accumulation in glia functions to sequester toxic oxidized fatty acids secreted by neurons under oxidative stress (Liu et al. 2017, 2015). Furthermore, blocking LD accumulation by inhibiting TG synthesis in LPS‐stimulated microglia increased the expression of proinflammatory cytokines, while inhibiting lipolysis reversed this effect (Li et al. 2023). This suggests that the role of LDs may be context‐dependent, as LDs formed by LPS stimulation show different volumes and distributions compared to LDs stimulated by linoleic acid and DHA (Tremblay et al. 2016; Khatchadourian et al. 2012).

Finally, the precise composition of lipid species within LDs in different contexts, such as normal aging, AD, stroke, TBI, and so on, has not yet been thoroughly investigated. While TGs and CEs are the major lipid classes typically associated with LDs, other lipid species—such as diacylglycerols, all‐*trans* retinyl esters, fatty acid esters of hydroxy fatty acids, and acyl ceramides—have also been reported in the LD lipidome of peripheral cells (Senkal et al. 2017; Wölk and Fedorova 2024; Riecan et al. 2022; Patel et al. 2022). Therefore, alterations in the composition of LDs may serve as useful indicators of dysregulated cellular homeostasis. The mechanisms by which these changes in LD composition influence microglial functions need further investigation.

## 3 Analytical Tools to Elucidate Microglial Lipidome

Recent advances in mass spectrometry methodologies have been largely responsible for the rapid propulsion of the lipidomics field. Specifically, mass spectrometry‐based experiments facilitate lipid detection, identification, and quantification by a combination of accurate mass‐to‐charge (*m*/*z*) measurements and tandem mass spectrometry (MS/MS). Lipid extraction from biological samples is a crucial first step in any lipidomics experiment. Total lipids can be extracted from isolated microglial cells from fresh brain tissue using Bligh and Dyer (1959) or Folch extraction (Folch, Lees, and Sloane Stanley 1957; Folch et al. 1951) protocols wherein lipids are solubilized in organic solvents like chloroform and methanol. Additional lipid extraction protocols have been explored, particularly for human plasma samples, though these approaches have yet to be extended to microglial lipid extraction (Saini et al. 2021).

There are two general MS‐based lipidomics strategies: targeted and non‐targeted. Briefly, a targeted approach seeks to identify and quantify a particular lipid or group of designated lipid species in a cellular or tissue extract. In contrast, global or non‐targeted lipidomics approach aims to define all lipids within a given sample qualitatively and quantitatively. Given our limited understanding of the microglial lipidome, global non‐targeted approaches will provide insights into alterations in lipid profiles before doing targeted lipidomics. To this end, techniques like multiple reaction monitoring (MRM) profiling (Xie et al. 2021; Ferreira et al. 2016) facilitate lipid screening based on variations in lipid functional groups with a goal to cover a larger discovery space for lipidomics screening. Importantly, MRM profiling can be utilized without chromatographic separation, thereby reducing the amount of time and sample volume required for discovery analysis. Recently, our group employed flow injection MRM profiling to screen for over 700 individual metabolites and over 1500 different lipid species encompassing 10 lipid classes in astrocytes, making it one of the largest lipidomic screens on glial cells to date (Guttenplan et al. 2021). Furthermore, the development of single cell lipidomics methods by incorporating a combination of mass spectrometry, chemical conjugation strategies, and automated data processing will enhance our knowledge about the depth and scale of lipid changes occurring in microglia at a given condition (Randolph et al. 2023).

Coupling the lipidomic characterization of microglia along with their transcriptomic, proteomic, and metabolomic changes will serve as a powerful tool to identify new molecular markers of microglial cell states and functions in AD, PD, MS, traumatic brain injury, and other neurological diseases. In addition to characterizing changes to lipid composition and changes to metabolism using bulk and single cell lipidomics, it is also useful to resolve the spatial characteristics of lipids in the CNS particular tissue. Spatial lipidomics using matrix‐assisted laser desorption/ionization (MALDI) imaging mass spectrometry (MSI) followed by immunohistology is quickly becoming an attractive approach to map the spatial information of lipids to individual cells in the CNS. Additionally, imaging LDs in rodent and post‐mortem human tissue is another method which can help us characterize cell health and metabolism in different disease paradigms. High‐resolution confocal microscopy like label‐free stimulated Raman scattering (SRS) microscopy (Zhang and Aldana‐Mendoza 2021) allows us to visualize lipid‐rich compartments without the need for any dye or antibody labeling steps. The need for such analytical tools and techniques to characterize microglial lipidome will only grow in the coming years.

## 4 Conclusions, Future Perspectives, and Outstanding Questions

Lipids are a diverse group of small molecules serving as the building blocks of all cellular membranes, providing structural stability, motility, and flexibility to cells. They also act as signaling molecules and regulate several metabolic processes essential for normal cell function. In this review, we have highlighted the various roles that different lipids play in regulating microglial cell states and functions. Microglial lipid metabolism undergoes the most significant changes during injury and disease. For example, lipids involved in key metabolic pathways have been identified as biomarkers of disease pathology in AD, PD, stroke, and other pathologies (Liu et al. 2021; Su et al. 2021; Tajima et al. 2013; Lambert et al. 2013). In Table 1, we provide a summary of the main lipid classes and specific subclasses associated with microglial function, the contexts and model systems used to identify these lipid mechanisms, and the potential genes and proteins identified as targets for further investigation of these processes. In Table 2, we highlight the key genes associated with specific lipid metabolism pathways that are identified broadly across all cell types. These genes can serve as new targets that may be further explored to assess microglial function by the research community.

One of the earliest associations between glial cells and lipids in the context of disease was made by Alois Alzheimer, who, while observing the brain tissue of a dementia patient, identified “glial cells with large adipose saccules” surrounding the Aβ plaques—what we now understand to be TG‐rich LDs in glia. Over a century later, we are only beginning to unravel how lipids modulate microglial function in AD and other neurodegenerative and neurological disorders (Prakash et al. 2023; Claes et al. 2021). Notably, several AD‐risk genes identified in recent genome‐wide association studies are specifically enriched in microglia and are involved in LDs and lipid metabolism, including *TREM2*, *ABCA7*, *APOE*, *CLU*, and *INPP5D*. In Table 3, we highlight several studies involving LD‐laden microglia in AD and other diseases. It provides details on the identification of LD^+^ microglia, the species and specific CNS regions where they were observed, the mechanisms through which LDs were formed in microglia, key targets identified for modulating the function of LD^+^ microglia, and the analytical tools used for their analysis. We hope that this information will help identify robust strategies for further research on LD‐laden microglia and drug discovery.

Much of what we know about microglial lipids has been translated from studies conducted in peripheral cells, particularly in macrophages. Despite their different origins—microglia originating from the embryonic yolk sac precursors and macrophages from bone marrow‐derived stem cells—they share several functional similarities (Li and Barres 2018). Given that much work remains in exploring specific lipid classes in microglia, including phospholipid subclasses such as PCs, PIs, and PGs, we have drawn on examples from macrophage studies to provide a broader understanding of how these lipids can modulate cell state and function. Additionally, the limited findings involving certain lipids have often been derived from studies using *in vitro* cell culture models. We advise caution when interpreting findings from cultured microglial cells, especially those in serum‐containing media, as these conditions may not accurately reflect the physiological *in vivo* state of microglia (Deng, Kersten, and Stienstra 2023). Therefore, future studies should prioritize investigating these lipids using relevant *in vivo* contexts.

Given the current knowledge, one can appreciate the vastness of the microglial lipid landscape and the added complexity due to the immense structural diversity of lipids. Hundreds, if not thousands, of lipids remain unidentified, forming what is referred to as the “dark lipidome.” Some of these currently unidentified lipids may play roles in critical disease‐related metabolic pathways and could serve as biomarkers of disease pathology (Su et al. 2021). Therefore, in addition to evaluating changes in individual lipid species during disease, it is essential to characterize the global lipidomic landscape of microglia. Understanding microglial cell states from a lipid‐centric perspective may hold the key to addressing several unanswered questions regarding microglial contributions and involvement in neurological and neurodegenerative disorders.

There is growing interest in strategies to profile the lipidomic states of microglia in response to various inflammatory triggers, such as injury and disease. Advances in high‐throughput mass spectrometry methodologies, which enable the identification of specific cellular and secretory lipids as well as the entire lipidome, will be fundamental to understanding microglial‐lipid‐mediated mechanisms (Randolph et al. 2023). Manipulating microglial lipids to influence cellular states and, ultimately, disease pathology will be a crucial focus in the coming years. Achieving this will require a comprehensive understanding of both known and unknown lipid classes, their structural properties, and their roles in microglial regulation across different contexts and conditions. Such knowledge is essential for developing lipid‐specific therapeutic approaches to modulate microglial cell states and functions in a context‐specific manner.

### 4.1 Outstanding Questions

1. How does the microglial lipidome change in different acute and chronic inflammatory environments and diseases?
2. How does the microglial lipidome vary across species?
3. How does the microglial lipidome change during development?
4. Are there sex‐specific differences in the microglial lipidome, and do these differences contribute to sex‐specific responses in injury and disease?
5. In what way does microglial lipid accumulation contribute to cell dysfunction and disease pathogenesis?
6. What are the specific classes and species of lipids that drive microglia‐mediated disease pathology?
7. How can we target microglial lipids or lipid‐related pathways to modulate cell function and identify new therapeutic targets for disease?

## Author Contributions

Conceptualization: P.P., G.C. Writing – original draft: P.P., C.E.R. and K.A.W. Writing – review and editing: P.P., C.E.R., K.A.W., and G.C. Supervision and Funding acquisition – G.C.

## Conflicts of Interest

G.C. is the Director of the Merck‐Purdue Center funded by Merck Sharp & Dohme, a subsidiary of Merck and the co‐founder of Meditati Inc. and BrainGnosis Inc. The remaining authors declare no competing interests.

## Data Availability

Data sharing is not applicable to this article as no new data were created or analyzed in this study.
